# Orthopedic Resident and Patient Perception of Electronic Medical Record Use During the Clinic Visit

**DOI:** 10.7759/cureus.43885

**Published:** 2023-08-21

**Authors:** Chris Rwigema, William H Fang, Xiao Chen, Christina Lane, Ian A Jones, C. Thomas Vangsness

**Affiliations:** 1 Department of Orthopaedics, University of Southern California Keck School of Medicine, Los Angeles, USA; 2 Department of Translational Medicine, Western University of Health Sciences, Los Angeles, USA; 3 Southern California Clinical and Translational Science Institute, University of Southern California Keck School of Medicine, Los Angeles, USA; 4 Department of Anesthesiology and Pain Medicine, University of Washington, Seattle, USA

**Keywords:** orthopedic resident physicians, computer use, satisfaction, patient perception, electronic medical records

## Abstract

Background

The transition from paper charts to electronic medical records (EMRs) has resulted in greater efficiency and reduced medical errors. This study aimed to examine the perception of patients and orthopedic residents regarding computer use during the clinic visit.

Methodology

This study utilized a cross-sectional cluster design. Orthopedic resident physicians were given a one-time general pre-visit survey. Additional surveys were given to patients and resident physicians post-visit. Surveys included questions that assessed satisfaction and the perceived impact of computer usage on doctor-patient interactions. Logistic generalized estimating equations were run to determine if there was an association between patient response and clinician assessment, adjusting for repeated measures within clinicians.

Results

A total of 80 patients and 15 residents completed the surveys. Results from the physician pre-visit survey showed that more residents perceived the computer as having a “negative” (47%) than “positive” (26%) effect on their relationship with patients. According to the post-visit analysis, patients perceived the residents’ use of the EMR as having an overall positive effect on their ability to establish a personal connection and having a positive effect on their ability to give them attention.

Conclusions

Overall, there was little correlation between patient and resident perception of the computer’s effect on their relationship. Patients generally perceived the computer as having a positive effect on their interaction with the residents even when residents had a negative perception of the computer’s effect on their interaction.

## Introduction

The healthcare industry has undergone a massive transition from paper charts to electronic medical records (EMRs). These changes will improve the overall quality of healthcare by improving efficiency, reducing medical errors, and curbing healthcare expenditures. The benefits of computerization include comprehensive documentation of a patient’s medical history [1,2], easy access to medical data from remote sites [2], improved communication among the various providers involved in health care, access to state-of-the-art resources over the Internet, and clinical decision support [3,4].

There are concerns regarding security, confidentiality, time incurred by EMR use, and negative impacts on the quality of patient care [5,6]. Computer use can affect physician-patient communication. Supporting this theory, Booth et al. demonstrated that these increasingly complex computer tasks monopolized the doctor’s attention, which was detrimental to the patient-doctor relationship [7]. Shachak et al. suggested that patient-doctor communication was perhaps the most significant component of a healthcare visit, with breaks in communication leading to worse health outcomes [4]. The use of the computer can lead to a decrease in eye contact and lowered sensitivity to patient responses because of increased distractions [8]. Effective patient-doctor communication can strengthen trust, improve patient adherence, and ultimately lead to better patient outcomes [9].

Hollenbeck et al. studied the effect of EMRs on efficiency while gathering information on resident attitude [10]. The use of EMRs was highly accurate and was more likely to contain pertinent clinical information. However, survey results showed that patients were more likely to agree that computer usage adversely affected the amount of time the resident physician spent talking, looking, and examining during the visit. Christino et al. examined the relationship between time spent on clinical documentation versus time available for patient care [11]. The study showed that most resident physicians from 24 different specialties (including orthopedics) reported “feeling rushed and frustrated because of documentation demands.” Both of these studies documented the negative impacts of the implementation of EMRs.

Medical documentation and clerical tasks have seen an increase in the past two decades, taking away time spent on direct patient care and communication. There has been a rising focus on greater documentation leading to increased time pressure and resident dissatisfaction [12]. Oxentenko et al. reported that of the 16,402 surveyed residents, 67.9% spent over four hours per day on documentation [13]. Other studies have shown residents spending as little as 65 minutes a day [14]. Given the potential positive influence of EMRs on daily hospital activities, quantifying resident perceptions and patient satisfaction may provide valuable information regarding the use of the computer and EMRs during the clinic visit.

This study aimed to examine the impact of the office computer on the orthopedic physician-patient interaction. The study compared resident and patient perceptions of exam room computer use in a large teaching hospital. The goal of this study was to assess the resident and patient perceptions of the exam room computer use and its impact on doctor-patient communication.

## Materials and methods

Study setting and design

The study was conducted in accordance with the relevant guidelines and regulations of the University of Southern California (USC) and was approved by the ethical committee (IRB Exempt, #HS-16-00548) This study utilized a cross-sectional design at an academic orthopedic clinic with patients seeing one of 15 different orthopedic residents over 12 months. Participation was voluntary for both residents and patients. Patients under the age of 18 years were excluded from the study. Orthopedic residents and clinic patients were given a short information sheet and consent form to read and sign before the beginning of the study.

Data collection

General demographic information such as gender, ethnicity, race, and the highest level of education was obtained from patients. Direct personal identifiers were removed from both resident (coded) and patient (de-identified) data to deidentify participants. Patient and resident physician perception survey questions were compiled from the literature [5,15,16]. Residents were given a one-time general survey before the first patient visit consisting of eight questions assessing their baseline computer skills and their initial opinions on computer/EMR usage in their practice (Appendices). Post-visit surveys with specific statements about the satisfaction and the impact of stationary computer use on patient-doctor communication were then given to patients and residents (Appendices).

The resident post-visit survey consisted of three questions specific to each patient visit assessing how the resident felt about the patient visit and measuring the effect of the computer on the visit. The post-visit patient survey consisted of 10 questions that assessed how they perceived the effect of the computer during their visit. A standard five-point Likert scale was used in both surveys, with responses ranging from “very positive” to “very negative” (“no effect” response in the middle).

All data sheets were deidentified and entered into a REDCap database hosted at Keck School of Medicine USC [17,18]. REDCap is a web-based application designed for developing databases and supporting data capture for research studies. Each patient in the study was required to complete only one survey. Spanish versions of surveys were provided for Spanish-speaking patients.

Statistical analysis

Descriptive statistics for all items were created to examine the ordinal scale distribution for all items. Due to sparse distribution across the five Likert categories, combinations were made for adequate representation in the cells. For statistical measurement, resident and patient answers of “Very positive” and “Positive” were combined into “Positive.” “Very negative” and “Negative” responses were combined into “Negative.” The association between patient demographics and ratings was explored using Spearman correlations and the chi-square tests, as applicable. The association of resident ratings and patient ratings was computed using generalized estimating equations to allow for the nested data structure. The dependent variable was the dichotomized patient response (positive versus no effect/negative) to each post-visit item. The primary independent variable was the three-level physician response (positive, neutral, and negative) on each of the three post-visit items. Effect sizes were estimated to describe the size of the effect. Analyses were performed using SPSS Statistics for Windows version 24 (IBM Corp., Armonk, NY, USA). P-values <0.05 were considered to indicate statistically significant differences.

## Results

A total of 80 patients completed the post-visit survey (Table 1). Gender was fairly distributed with 53% (N = 42) males. The majority of patients were Hispanic (73%, N = 58) and white (23%, N = 18); 68% (N = 54) had a high school/GED level education and lower. Of the 15 resident physicians who participated in the study, 14 were male. The number of surveys each resident physician filled out is reported in Table 2. No other demographic data were collected for the residents.

**Table 1:** Surveyed patient demographics.

		N	%
Gender	Male	42	52.5
	Female	38	47.5
Education	Did not attend high school	9	11.3
	Some high school	16	20
	High school diploma/GED	29	36.3
	Associate degree	10	12.5
	Bachelor’s degree	13	16.3
	Master’s, doctorate, or professional degree	3	3.8
Ethnicity	Hispanic	58	72.5
	Non-Hispanic	22	27.5
Race	White	18	22.5
	Black or African American	7	8.8
	Asian	7	8.8
	Other	48	60

**Table 2:** Resident physician characteristics.

Physician gender	Count	% of total
Male	14	93%
Female	1	7%
	Number of completed surveys	% of total
B	13	16%
L	13	16%
K	7	9%
M	7	9%
A	6	8%
E	5	6%
N	5	6%
H	4	5%
I	4	5%
J	4	5%
D	3	4%
G	3	4%
C	2	3%
F	2	3%
O	2	3%

Physician pre-visit survey

The physician pre-visit survey (Table 3) showed that more residents perceived using a computer as having a more “negative” (47%) than “positive” (26%) effect on their relationship with patients. Additionally, a majority of the residents also felt the use of computers in the clinic room would have a negative impact on both their ability to communicate with patients (53%) as well as their ability to maintain eye contact with patients (87%). Despite these perceptions, 67% reported the EMR/computer having a “positive” effect on their efficiency.

**Table 3:** Resident pre-visit survey results.

		Very positive	Positive	No effect	Negative	Very negative
1. Using a computer in the exam room has had a ___ effect on my relationship with my patients	N	2	2	4	7	0
	%	13%	13%	27%	47%	0%
2. Using a computer in the exam room has had a ____ effect on my ability to communicate with my patients	N	2	5	0	8	0
	%	13%	33%	0%	53%	0%
3. Using a computer in the exam room has had a ____ effect on my ability to maintain eye contact with patients	N	0	1	1	10	3
	%	0%	7%	7%	67%	20%
4. Using a computer in the exam room has had a ____ effect on the time I have available to perform the physical examination	N	1	4	6	4	0
	%	7%	27%	40%	27%	0%
5. Using a computer in the exam room has had a ____ effect on my ability to give attention to patients	N	1	0	4	10	0
	%	7%	0%	27%	67%	0%
6. Using a computer in the exam room has had a ____ effect on my efficiency	N	2	8	2	3	0
	%	13%	53%	13%	20%	0%
7. Using a computer in the exam room has had a ____ effect on the quality of my residency training experience	N	2	3	7	3	0
	%	13%	20%	47%	20%	0%
8. Using a computer in the exam room has had a ____ effect on my ability to listen to patients	N	0	1	9	5	0
	%	0%	7%	60%	33%	0%

To determine if patient comfort or perception differed based on clinician assessment, logistic generalized estimating equations were performed. Each of the three resident post-survey responses was compared to each of the 10 patient post-visit survey responses. There were 20 patients who were assessed by a resident with a positive perception, 31 patients who were assessed by a neutral resident, and 29 patients who were assessed by residents with a negative perception.

Table 4 shows the correlation between how the residents felt post-visit regarding the effect of the computer and the satisfaction of the visit. For the post-visit analysis, patients perceived the residents’ use of the computer as having a positive effect on their relationship for all levels of the residents’ computer perception. Residents who perceived using a computer would have a positive impact (70.0%) or a neutral impact (42.4%) were correlated to the patient feeling that the use of a computer in the exam room had a more positive effect on the ability to establish a personal connection with the provider (p = 0.003).

**Table 4:** Correlating post-visit patient survey with Q1 of post-visit physician satisfaction. P < 0.05 was considered statistically significant.

		S1. Using a computer had a ____ impact on how satisfied I was with this visit						
		Positive		Neutral		Negative		
		N	%	N	%	N	%	P-value
Q1. How comfortable are you using computers for work, social media, email, shopping, etc.?	Comfortable	31	77.5%	20	60.6%	5	71.4%	0.25
	Neutral/not comfortable	9	22.5%	13	39.4%	2	28.6%	
Q2. The doctor’s use of the computer during this visit had the following effect on our relationship	Positive	34	85.0%	19	57.6%	6	85.7%	0.08
	No effect/negative	6	15.0%	14	42.4%	1	14.3%	
Q3. The doctor’s use of the computer during this visit had the following effect on our communication	Positive	32	80.0%	21	63.6%	5	71.4%	0.36
	No effect/negative	8	20.0%	12	36.4%	2	28.6%	
Q4. The doctor’s use of the computer had a _____ effect on their ability to maintain eye contact with me	Positive	25	62.5%	17	51.5%	4	57.1%	0.67
	No effect/negative	15	37.5%	16	48.5%	3	42.9%	
Q5. The doctor’s use of the computer had a _____ effect on their ability to give me attention	Positive	30	75.0%	15	45.5%	4	57.1%	0.07
	No effect/negative	10	25.0%	18	54.5%	3	42.9%	
Q6. The doctor’s use of the computer had a ____ effect on their ability to listen to me	Positive	27	67.5%	14	42.4%	4	57.1%	0.08
	No effect/negative	13	32.5%	19	57.6%	3	42.9%	
Q7. The doctor’s use of the computer had a ____ effect on my overall satisfaction with this visit	Positive	31	77.5%	20	60.6%	7	100.0%	0.20
	No effect/negative	9	22.5%	13	39.4%	0	0.0%	
Q8. The doctor’s use of the computer had a ____ effect on my ability to establish a personal connection with him/her	Positive	28	70.0%	14	42.4%	6	85.7%	0.003*
	No effect/negative	12	30.0%	19	57.6%	1	14.3%	
Q9. The doctor’s use of the computer had a ____ effect on their efficiency	Positive	30	75.0%	20	60.6%	5	71.4%	0.49
	No effect/negative	10	25.0%	13	39.4%	2	28.6%	
Q10. What is your opinion on how much time the doctor spent on the computer during this visit?	Comfortable	37	92.5%	27	81.8%	7	100.0%	0.20
	Neutral/not comfortable	3	7.5%	6	18.2%	0	0.0%	

There were no statistically significant results (p > 0.05) between residents’ opinions on how much time they spent on the computer during the visit and the post-visit patient survey.

Table 5 shows the correlation between the post-visit patient survey and how the residents felt about the computer impacting their ability to connect with patients. When residents perceived the computer as having a positive or negative effect on their ability to connect with patients, patients still perceived the residents’ use of the computer as having a positive effect on their ability to listen and establish a personal connection (p < 0.001). Patients felt that the use of the computer in the exam room had a positive impact on their relationship (p = 0.02) and on communications (p = 0.001), despite the resident’s perception that the computer had a negative impact on their ability to connect with patients. When residents perceived the computer as having a positive or negative effect on their ability to connect with patients, patients still perceived the residents’ use of the computer as having a positive effect on their ability to give them attention (p = 0.001). When residents were neutral about their perception, patients either responded with “no effect” or “negative” 58% and 65% of the time, respectively (p = 0.03 and <0.001, respectively).

**Table 5:** Correlating post-visit patient survey with Q2 of post-visit physician connection survey. P < 0.05 was considered statistically significant.

		S2. Using a computer had a ____ effect on my ability to connect with the patient						
		Positive		Neutral		Negative		
		N	%	N	%	N	%	p
Q1. How comfortable are you using computers for work, social media, email, shopping, etc.?	Comfortable	16	80.0%	18	58.1%	22	75.9%	0.09
	Neutral/not comfortable	4	20.0%	13	41.9%	7	24.1%	
Q2. The doctor’s use of the computer during this visit had the following effect on our relationship	Positive	19	95.0%	18	58.1%	22	75.9%	0.02*
	No effect/negative	1	5.0%	13	41.9%	7	24.1%	
Q3. The doctor’s use of the computer during this visit had the following effect on our communication	Positive	18	90.0%	19	61.3%	21	72.4%	0.001*
	No effect/negative	2	10.0%	12	38.7%	8	27.6%	
Q4. The doctor’s use of the computer had a _____ effect on their ability to maintain eye contact with me	Positive	14	70.0%	14	45.2%	18	62.1%	0.10
	No effect/negative	6	30.0%	17	54.8%	11	37.9%	
Q5. The doctor’s use of the computer had a _____ effect on their ability to give me attention	Positive	17	85.0%	13	41.9%	19	65.5%	0.001*
	No effect/negative	3	15.0%	18	58.1%	10	34.5%	
Q6. The doctor’s use of the computer had a ____ effect on their ability to listen to me	Positive	14	70.0%	13	41.9%	18	62.1%	0.03*
	No effect/negative	6	30.0%	18	58.1%	11	37.9%	
Q7. The doctor’s use of the computer had a ____ effect on my overall satisfaction with this visit	Positive	17	85.0%	20	64.5%	21	72.4%	0.001*
	No effect/negative	3	15.0%	11	35.5%	8	27.6%	
Q8. The doctor’s use of the computer had a ____ effect on my ability to establish a personal connection with him/her	Positive	16	80.0%	11	35.5%	21	72.4%	<0.001*
	No effect/negative	4	20.0%	20	64.5%	8	27.6%	
Q9. The doctor’s use of the computer had a ____ effect on their efficiency	Positive	16	80.0%	20	64.5%	19	65.5%	0.10
	No effect/negative	4	20.0%	11	35.5%	10	34.5%	
Q10. What is your opinion on how much time the doctor spent on the computer during this visit?	Comfortable	18	90.0%	26	83.9%	27	93.1%	0.39
	Neutral/not comfortable	2	10.0%	5	16.1%	2	6.9%	

## Discussion

This study assessed whether there was an association between patients’ and orthopedic residents’ perceptions of computer use and their communication. Overall, there was little association between patient and resident perception of the EMR/computer’s effect on their relationship. Patients generally perceived computer usage as having a positive effect on their interaction with the orthopedic residents, as well as their personal ability to establish a connection with the provider. This held true even when the residents had a negative perception of the computer’s effect on their interaction. No statistically significant difference (p > 0.05) was found in the perception of eye contact, residents’ efficiency, or perception of time spent on EMR during visits between patients and physicians. In general, more residents perceived the use of the computer during visits as having a more negative effect on their relationship and their ability to communicate with patients. However, the majority of residents perceived EMR usage as having a positive effect on their efficiency during visits.

When residents perceived computer use as positively impacting satisfaction or the ability to connect with patients, there was a higher proportion of patients responding with positive answers. However, when residents had a greater negative outlook on computers, patients still perceived the resident’s use of the computer as having a positive effect on the clinic visit. A possible explanation might be that residents who have negative views feel they have to compensate for their negative perceptions by giving more attention and care to the patients. Residents who were neutral about their perception had more patients answering no effect or having a negative outlook on how the computer affected their clinic visit. These residents might not have conscientiously attempted to improve communication, affecting their clinic visits and patient-physician interaction.

This data, and a review of the literature, permit no definitive conclusions about whether computers have a negative effect on the overall doctor-patient interaction. The literature has mixed results regarding how the computer affects physician-patient interaction. Patients expressed frustration when physicians were not transparent with their computer use and felt the use of the computer led to decreased quality of care [19]. Rouf et al. showed that patients seen by residents were more likely to agree that the computer adversely affected the amount of time physicians spent talking to, looking at, and examining them compared to those seen by faculty physicians [15]. Residents were more likely to report negative perceptions compared to the faculty [20]. The faculty were less likely to report that the presence of the computer interfered with their interaction with the patients. Similarly, patients seen by faculty physicians were less likely to report that communication was compromised by the presence of the computer. However, none of the differences between residents and faculty were found to be statistically significant [20]. These findings introduce the possibility that with more experience, physicians may be able to overcome potential communication barriers caused by the presence of the computer.

Other studies have shown more neutral results regarding the effect of EMR on communication. Rethans et al. analyzed whether computers in the exam room caused doctors to appear less personal to patients and found that the majority of patients believed that contact with their doctor was as easy as before the implementation of the computer [21]. Makoul et al. found that even though there was no significant difference in visit times with or without a computer, physicians took a more proactive stance in clarifying information using the computer [22]. The presence of a computer changes the verbal, visual, and postural connection between a physician and a patient [23]. It is important to consider the physical placement of the computer and monitor.

Study limitations

In this study, only resident physicians were questioned regarding their perception of the impact that the computer had on their interaction with the patient. Although there is a possibility that surveying attending faculty with more experience may yield different results, the reliance on EMR systems is relatively new and no assumptions can be made. Resident baseline computer skills were not evaluated in the study design as well, leading to potential bias in how comfortable each resident felt about the computer. The surveys used in this study were non-validated and were prepared by author consensus. These non-validated surveys may be subject to measurement error, and conclusions cannot be drawn with total confidence.

This study required voluntary participation, which could lead to bias in survey responses. The sample size was small and only one female resident physician was included in the study, making the results difficult to generalize. Additionally, there may be a bias in physicians’ behaviors when interacting with patients due to prior knowledge that a survey would be conducted after each visit. The study was conducted in only one clinic, and further comparison studies need to be implemented at other clinics to fully understand the influence of the computer on physician-patient interaction.

Lastly, the physical location of the stationary computer and the patient’s position in the exam room was not evaluated. The location of the computer could impact the resident’s eye contact and body positioning (i.e., facing to the side or away from the patient) during the visit, which may lead to patients feeling more negative about their visit. Additionally, the effects between the interpreter and non-English-speaking patients were not evaluated.

## Conclusions

Our study reveals that there was little association between patient and resident perception of the EMR/computer’s impact on their medical visit dynamics. In general, patients viewed computer usage positively, finding that it had a beneficial effect on their interactions with orthopedic residents and helped establish a more meaningful connection with their healthcare provider. Similarly, a majority of residents held a positive impression of using computers during visits, primarily due to the improved efficiency it brought. Although some concerns related to communication issues emerged, they were not statistically significant. For future guidance, it is crucial to focus on training physicians in the integration of patient-centered communication strategies while utilizing the EMR in clinical practice, including fostering active listening and incorporating the use of EMR systems in a patient-oriented manner.

This study’s implications extend to the future design of communications curricula and the content of required EMR training within healthcare facilities. Based on our findings and identified best practices, we propose the development of a patient-centered EMR use curriculum for both faculty and trainees. This curriculum should encompass training on utilizing the EMR to promote teamwork among healthcare professionals, maximizing patient engagement and education by collaboratively reviewing health information, sharing the screen to promote transparency, and optimizing eye contact and body positioning. By implementing such a curriculum, we can enhance the overall patient experience and improve the quality of care delivered through EMR usage.

## Appendices

**Table 6:** Pre-visit resident survey.

	1	2	3	4	5
1. Using a computer in the exam room has had a _____ effect on my relationship with my patients					
	Very positive	Positive	No effect	Negative	Very negative
2. Using a computer in the exam room has had a _____ effect on my ability to communicate with patients					
	Very positive	Positive	No effect	Negative	Very negative
3. Using a computer in the exam room has had a _____ effect on my ability to maintain eye contact with patients					
	Very positive	Positive	No effect	Negative	Very negative
4. Using a computer in the exam room has had a _____ effect on the time I have available to perform the physical examination					
	Very positive	Positive	No effect	Negative	Very negative
5. Using a computer in the exam room has had a _____ effect on my ability to give attention to patients					
	Very positive	Positive	No effect	Negative	Very negative
6. Using a computer in the exam room has had a _____ effect on my efficiency					
	Very positive	Positive	No effect	Negative	Very negative
7. Using a computer in the exam room has had a _____ effect on the quality of my residency training experience					
	Very positive	Positive	No effect	Negative	Very negative
8. Using a computer in the exam room has had a _____ effect on my ability to listen to patients					
	Very positive	Positive	No effect	Negative	Very negative

**Table 7:** Post-visit patient survey.

	1	2	3	4	5
1. How positive are you using computers for work, social media, email, shopping, etc.?					
	Very positive	Positive	No effect	Negative	Very negative
2. The doctor’s use of the computer during this visit had the following effect on our relationship					
	Very positive	Positive	No effect	Negative	Very negative
3. The doctor’s use of the computer during this visit had the following effect on our communication					
	Very positive	Positive	No effect	Negative	Very negative
4. The doctor’s use of the computer had a _____ effect on their ability to maintain eye contact with me					
	Very positive	Positive	No effect	Negative	Very negative
5. The doctor’s use of the computer had a _____ effect on their ability to give me attention					
	Very positive	Positive	No effect	Negative	Very negative
6. The doctor’s use of the computer had a _____ effect on their ability to listen to me					
	Very positive	Positive	No effect	Negative	Very negative
7. The doctor’s use of the computer had a _____ effect on my overall satisfaction with this visit					
	Very positive	Positive	No effect	Negative	Very negative
8. The doctor’s use of the computer had a _____ effect on my ability to establish a personal connection with him/her					
	Very positive	Positive	No effect	Negative	Very negative
9. The doctor’s use of the computer had a _____ effect on their efficiency					
	Very positive	Positive	No effect	Negative	Very negative
10. What is your opinion on how much time the doctor spent on the computer during this visit					
	They spent too much time on the computer	They spent the right amount of time	They did not spend enough time on the computer	They spent a lot of time on the computer	They spent too much time on the computer

**Table 8:** Resident post-visit survey.

	1	2	3	4	5
1. Using a computer had a _____ impact on how satisfied I was with this visit					
	Very positive	Positive	No effect	Negative	Very negative
2. Using a computer had a _____ effect on my ability to connect with the patient					
	Very positive	Positive	No effect	Negative	Very negative
3 What is your opinion on how much time you spent on the computer during this visit?					
	Very positive	Positive	No effect	Negative	Very negative
